# Supplementary material for: Panax Ginseng alleviates thioacetamide-induced liver injury in ovariectomized rats: Crosstalk between inflammation and oxidative stress
Source: PLoS One. 2021 Nov 29;16(11):e0260507. doi: 10.1371/journal.pone.0260507 (PMC8629276; doi:10.1371/journal.pone.0260507)
Supplement: S1 File — (DOC) [file pone.0260507.s002.doc]

Highlights

1. Ovariectomization of rats resulted in non-significant changes in liver function
2. Panax ginseng reduces oxidative stress & inflammation and improves liver function
3. Panax ginseng reduces circulating AGEs &TNF-α and thus spares liver tissue
4. Panax ginseng reduces MPO and NF-kβ liver immunohistochemical staining.
