# Supplementary figures and images for: Panax Ginseng alleviates thioacetamide-induced liver injury in ovariectomized rats: Crosstalk between inflammation and oxidative stress
Source: PLoS One. 2021 Nov 29;16(11):e0260507. doi: 10.1371/journal.pone.0260507 (PMC8629276; doi:10.1371/journal.pone.0260507)

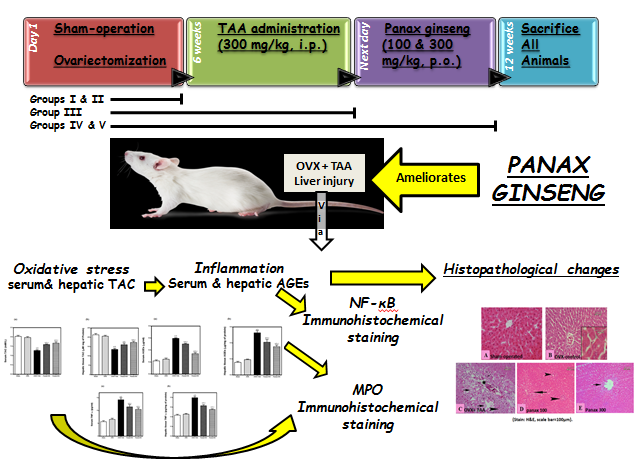

Supplement: S1 Graphical abstract — (TIF) [file pone.0260507.s003.tif]
