# Supplementary material for: Panax Ginseng alleviates thioacetamide-induced liver injury in ovariectomized rats: Crosstalk between inflammation and oxidative stress
Source: PLoS One. 2021 Nov 29;16(11):e0260507. doi: 10.1371/journal.pone.0260507 (PMC8629276; doi:10.1371/journal.pone.0260507)
Supplement: Attachment — Submitted filename: Response to reviewers comments.doc [file pone.0260507.s004.doc]

**Response to reviewers’ comments**

Manuscript number: **PONE-D-21-15006.**

Title: **Panax Ginseng Alleviates Thioacetamide-Induced Liver Injury in Ovariectomized Rats Via Modulation of Advanced Glycation End Products: Crosstalk between Inflammation and Oxidative Stress.**

We sincerely thank the editor and the respectable reviewers for considering our manuscript.

**Please find below a point to point response to the reviewers’ comments:**

All changes are marked in the main manuscript file via the track changes mode.

Comments to the authors

1. If the authors have adequately addressed your comments raised in a previous round of review and you feel that this manuscript is now acceptable for publication, you may indicate that here to bypass the “Comments to the Author” section, enter your conflict of interest statement in the “Confidential to Editor” section, and submit your "Accept" recommendation.

Reviewer #2: (No Response)

2. Is the manuscript technically sound, and do the data support the conclusions?
The manuscript must describe a technically sound piece of scientific research with data that supports the conclusions. Experiments must have been conducted rigorously, with appropriate controls, replication, and sample sizes. The conclusions must be drawn appropriately based on the data presented.

Reviewer #2: Partly

3. Has the statistical analysis been performed appropriately and rigorously?

Reviewer #2: Yes

4. Have the authors made all data underlying the findings in their manuscript fully available?
The [PLOS Data policy](http://www.plosone.org/static/policies.action#sharing) requires authors to make all data underlying the findings described in their manuscript fully available without restriction, with rare exception (please refer to the Data Availability Statement in the manuscript PDF file). The data should be provided as part of the manuscript or its supporting information, or deposited to a public repository. For example, in addition to summary statistics, the data points behind means, medians and variance measures should be available. If there are restrictions on publicly sharing data—e.g. participant privacy or use of data from a third party—those must be specified.

Reviewer #2: Yes

5. Is the manuscript presented in an intelligible fashion and written in standard English?
PLOS ONE does not copyedit accepted manuscripts, so the language in submitted articles must be clear, correct, and unambiguous. Any typographical or grammatical errors should be corrected at revision, so please note any specific errors here.

Reviewer #2: Yes

**6. Review Comments to the Author**

Reviewer #2:

In general, the authors did not address my previous concerns and did not seem to attempt to address some points. Furthermore there are still major flaws in the results of the paper, which would lead this reviewer to think that the stated conclusions are not sound. Without the extra time and effort put forth it appears as if the quality of this paper is lacking and it doesn't bring a significant amount of knowledge to the field.

Remaining comments:

- The authors refused to do the CK-19 staining. It is known that TAA induces Cholangiocyte damage, as well cholangiocytes are responsive to oestrogen signaling; therefore, this should have been performed.

**As the reviewer stated “It is known that TAA induces Cholangiocyte damage, as well cholangiocytes are responsive to oestrogen signaling”…Actually we clarified that TAA can indeed induce cholangiocarcinoma. And by the way, cholestatic injury is also apparent via the predominantly increased levels of alkaline phosphatase.**

**However, the study was not mainly concerned with testing cholangiocarcinoma. Rather, we were mainly concerned with demonstrating the relationship between AGEs, NF-kβ, TNF-α and MPO in the modulation of inflammation and oxidative stress-induced bt TAA in hepatic tissue.**

**So the required stain was out of the scope of this particular research….**

-ROS levels and antioxidant activities should be measured in the livers of Rats and added to Figure 2. Serum levels are fine but don't show changes in hepatic damage.

**Thank you for this valuable remark.**

**Hepatic tissue TAC, AGEs have been measured and added in the results section in figures 2 and 3 as per the reviewer’s comment.**

**Moreover, Serum and Hepatic tissue contents of TNF-α have been measured and added in results section in figure 4 to add more impact to the study.**

- H&E label should be labeled as H&E, as well as the names of the groups on the pictures so it is easier for the reader to understand the image. This labeling approach should be done for all figures.

**Thank you for this appreciated valuable remark.**

**The label as well as the names of the groups have been added in all images as per the reviewer’s comment.**

- If the authors are going to make comments on liver fibrosis (based on H&E staining) then this needs to be done with other methods that are more rigorous. Liver damage is associated with liver fibrosis; therefore, this should have been included in this paper and not in a separate study. Especially since it is known that oestrogen signaling influences HSC activation and liver fibrosis.

**It’s true that fibrotic changes have been noted on H&E by a professional histopathologist. However, our study was not mainly concerned with fibrosis.**

**we suggest further studies to assess this point in this experimental model in future researches and this suggestion has been added to the discussion section in the main manuscript.**

- The NFkB staining is still of poor quality - you cannot distinguish nuclear versus cytoplasmic expression.

**The immunohistochemical images’ quality has been further improved to pinpoint antibody staining. Please notice that immunohistochemical images are usually different from H&E images where the former are mainly concerned with outlining the protein expression rather than detailed features of the cells**

**Any professional histopathologist can differentiate the changes in such images.**

-As well, the addition of serum TNF-alpha is not specific to increased NFkB signaling in the liver; therefore, the authors did not rigorously evaluate downstream mechanisms as previously asked.

**We actually measured both serum and hepatic tissue TNF alpha as a signaling downstream pathway for NF-KB as per the reviewer’s previous comment. This data is added in figure 4.**

- The MPO staining is still not convincing even though the authors said they improved the quality?

**Images’ quality has been improved by a professional histopathologist.**

- The authors still did not add discussion on oestrogen signaling and ovariectomy in the introduction or the discussion even though the ovariectomy is performed to reduce oestrogen signaling?? They also still do not provide a rationale for looking at ginseng in females lacking oestrogen signaling?

**Both the introduction and the discussion sections contain reflections on the effect of estrogen deficiency on liver damage.**

**We stated clearly that “reductions in circulating estrogen levels that occur during menopause foster the conditions that increase the damage in the liver tissue. The Beneficial effects of estrogen in liver diseases include enhancement of innate immunity and antioxidant effects along with the promotion of mitochondrial function and DNA synthesis in hepatocytes. Estrogen also causes inhibition of fibrogenesis as well as reduction of release of oxidative stress parameters and inflammatory cytokines from Kupffer cells” in the introduction section. Therefore, post-menopausal females might display more severe liver disease progression.**

**Actually, the first page of the discussion section discusses the relationship between estrogen signaling and liver damage.**

**Moreover, we measured serum estradiol levels for all groups as per the reviewer’s comment and the results were added in Table 1. As expected, Ovariectomization resulted in a significant reduction in serum estradiol (E2) level as compared to sham-operated rats; signifying reproductive senescence in rats.**

- The ginseng did not significantly reduce liver damage in ovariectomy mice compared to ovariectomy alone; therefore, this reviewer still disagrees with the title and conclusions of the study. Loss of oestrogen alone reduces liver damage, so what is the usefulness of the ginseng?

**This is totally untrue…By careful reading of figures 2, 3, 4, and even pathology and immunohistopathological images. Ginsing significantly reduced liver damage in ovariectomy rats compared to OVX alone alone.**

- They are still lacking proper controls without the TAA alone treatment.

**In this work, we were mainly concerned with the impact of estrogen deficiency in postmenopausal females on the protection against liver injury, therefore we compared the results of TAA+OVX group with OVX only group.**

**Thank you**
