# Supplementary material for: Panax Ginseng alleviates thioacetamide-induced liver injury in ovariectomized rats: Crosstalk between inflammation and oxidative stress
Source: PLoS One. 2021 Nov 29;16(11):e0260507. doi: 10.1371/journal.pone.0260507 (PMC8629276; doi:10.1371/journal.pone.0260507)
Supplement: Attachment — Submitted filename: Response to reviewers comments.doc [file pone.0260507.s005.doc]

**Response to reviewers’ comments**

**Rebuttal letter**

Manuscript number: **PONE-D-21-25160.**

Title: **Panax Ginseng Alleviates Thioacetamide-Induced Liver Injury in Ovariectomized Rats Via Modulation of Advanced Glycation End Products: Crosstalk between Inflammation and Oxidative Stress.**

We sincerely thank the editor and the respectable reviewers for their precious time and constructive criticisms. Their valuable comments were of great help in revising the manuscript and they definitely improve it.

Accordingly, the revised manuscript has been systematically enhanced with new information and

additional interpretations. Following are the detailed responses to the journal requirements and

reviewers comments:

**Please find below a point to point response to the reviewers’ comments:**

All changes are marked in the main manuscript file via the track changes mode.

**Comments to the authors**

1. Please ensure that your manuscript meets PLOS ONE's style requirements, including those for file naming. The PLOS ONE style templates can be found at 
https://journals.plos.org/plosone/s/file?id=wjVg/PLOSOne_formatting_sample_main_body.pdf and 
<https://journals.plos.org/plosone/s/file?id=ba62/PLOSOne_formatting_sample_title_authors_affiliations.pdf>

**Journal requitements have been strictly followed**

2. We note that you have stated that you will provide repository information for your data at acceptance. Should your manuscript be accepted for publication, we will hold it until you provide the relevant accession numbers or DOIs necessary to access your data. If you wish to make changes to your Data Availability statement, please describe these changes in your cover letter and we will update your Data Availability statement to reflect the information you provide

**All data will be uploaded as one single EXCEL file into the online system**

**Data Availability statement has been changed in the cover letter.**

Reviewers' comments:

Reviewer's Responses to Questions

Comments to the Author

1. Is the manuscript technically sound, and do the data support the conclusions?

The manuscript must describe a technically sound piece of scientific research with data that supports the conclusions. Experiments must have been conducted rigorously, with appropriate controls, replication, and sample sizes. The conclusions must be drawn appropriately based on the data presented.

Reviewer #1: Yes

2. Has the statistical analysis been performed appropriately and rigorously?

Reviewer #1: Yes

3. Have the authors made all data underlying the findings in their manuscript fully available?

The [PLOS Data policy](http://www.plosone.org/static/policies.action#sharing) requires authors to make all data underlying the findings described in their manuscript fully available without restriction, with rare exception (please refer to the Data Availability Statement in the manuscript PDF file). The data should be provided as part of the manuscript or its supporting information, or deposited to a public repository. For example, in addition to summary statistics, the data points behind means, medians and variance measures should be available. If there are restrictions on publicly sharing data—e.g. participant privacy or use of data from a third party—those must be specified.

Reviewer #1: Yes

4. Is the manuscript presented in an intelligible fashion and written in standard English?

PLOS ONE does not copyedit accepted manuscripts, so the language in submitted articles must be clear, correct, and unambiguous. Any typographical or grammatical errors should be corrected at revision, so please note any specific errors here.

Reviewer #1: Yes

5. Review Comments to the Author

Please use the space provided to explain your answers to the questions above. You may also include additional comments for the author, including concerns about dual publication, research ethics, or publication ethics. (Please upload your review as an attachment if it exceeds 20,000 characters)

**Reviewer #1:**

In this work Mostafa et al evaluate the protective effects of Panax ginseng extract on thioacetamide (TAA) induced liver injury in ovariectomized rats, which mimic post-menopausal estrogen-deficient females. The authors find significant hepatoprotection at different levels, including antioxidant liver capacity, generation of AGEs, inflammation and injury. This study confirms the hepatoprotective and beneficial effects of Panax ginseng observed in previous works, including ovariectomized rats. There are some issues that need the authors attention.

1. It appears that the authors administered a single TAA injection, and evaluated liver injury-related parameters six weeks after this single dose. It is hard to believe that six weeks after that single TAA injection there are still signs of liver injury detectable. Upon acute liver injury rodents trigger a potent regenerative response. Other works using similar single doses of TAA report a rapid recovery of serum liver enzymes and bilirrubin, which almost normalize after one week (see for instance: PMID: 32901492). This aspect is critical and should be clarified.

**We would like to thank the reviewer for this question**

**Many research papers demonstrated that the use of single oral dose of TAA resulted in significant liver injury that last for more than 4 weeks post TAA adminstration.**

**Mostafa et al. (2017) demonstrated that a single intraperitoneal dose of TAA (300 mg/kg) resulted in significant hepatotoxic oxidative damage in rats after 30 days post TAA injection. The Histopathological examination of liver tissues showed significant hepatotoxicity as manifested by severe congestion of blood vessels, activated Kupfer cells, and enlarged hepatocytes with disappearance of sinusoids .**

**Waters *et al*. (2005) reported that** **TAA causes centrilobular liver necrosis following single dose administration, while repeated exposure results in bile duct proliferation along with hepatic cirrhosis .**

**Strnad *et al*. (2008****) reported that TAA caused more severe liver injury and fibrosis in mice than CCL-4 .**

**Of note, Wallace et al. (2015) reported that TAA is commonly used in the dose of 100-200 mg/kg, i.p. to induce long-lasting reproducible liver fibrosis that resembles alcoholic fibrogenesis with low mortality in experimental animals .**

**The administration of TAA via the oral or IP route is a safe and effective technique for the study of fibrosis in rodents. There is a wide variation in the route, dose, timing and animal used .**

Mostafa, R.E., Salama, A.A., Abdel-Rahman, R.F., Ogaly, H.A., 2016. Hepato-and neuro-protective influences of biopropolis on thioacetamide-induced acute hepatic encephalopathy in rats. Canadian Journal of Physiology and Pharmacology 95, 539-547.

Strnad, P., Tao, G.Z., Zhou, Q., Harada, M., Toivola, D.M., Brunt, E.M., Omary, M.B., 2008. Keratin Mutation Predisposes to Mouse Liver Fibrosis and Unmasks Differential Effects of the Carbon Tetrachloride and Thioacetamide Models. Gastroenterology 134, 1169-1179.

Wallace, M., Hamesch, K., Lunova, M., Kim, Y., Weiskirchen, R., Strnad, P., Friedman, S., 2015. Standard operating procedures in experimental liver research: thioacetamide model in mice and rats. Laboratory animals 49, 21-29.

Waters, N.J., Waterfield, C.J., Farrant, R.D., Holmes, E., Nicholson, J.K., 2005. Metabonomic deconvolution of embedded toxicity: application to thioacetamide hepato-and nephrotoxicity. Chemical research in toxicology 18, 639-654.

**Like wise in the current work, all measured parameters showed significant deterioration 6 weeks post TAA dose. The Histopathological examination of liver tissues showed noticeable fibrosis in portal area with focal aggregation of inflammatory cells and around the central vein. Vacuolar degeneration and karyolysis could be seen clearly in many hepatocytes along with aggregations of inflammatory cells and congestion of main blood vessels.**

2. A recent study (Kobilhova E, et al Res. 2020: PMID: . Physiol. 32901492) demonstrated the lack of effect of ovariectomy on TAA-induced acute liver injury in rats. This previous publication is directly relevant to the present report and should be cited and discussed.

**We sincerely thank the reviewer for pointing to this study.**

**Actually our data is in-line with this work since ovariectomized rats showed a non-significant change in the measured parameters**

**The aforementioned study has been cited and discussed in the revised manuscript**.

3. In Fig. 5 the authors discuss about the degree of liver fibrosis found in tissue sections from the different groups of rats. Fibrosis needs to be directly assessed, at least by Sirius Red staining, and quantitated.

**Thank you for this valuable comment.**

**Liver fibrosis has been assessed and quantified as per the reviewer comment.**

**Masson trichrome staining has been used for this assessment since Sirius Red staining is not available in our lab.**

**Masson trichrome staining is frequently and accurately used for assessment of fibrosis.**

**This data is added in figure 6 and the quantified results have been added in table 2.**

4. In Fig. 6, immunohistochemistry for Nf-KB should also be quantitated. The Nf-KB subunit examined and the reference of the commercial antibody used should be provided.

**We sincerely thank the reviewer for this valuable remark**

**The Nf-KB subunit examined (Anti-NF-kB p65) and the reference of the commercial antibody (ab16502, Abcam, UK) have been added to materials and methods section in page 8.**

**Moreover, the immunohistochemistry for Nf-KB p65 was quantitated and added in results section in table 3**

5. In Fig. 7, immunohistochemistry for MPO should also be quantitated. The reference of the commercial antibody used should be provided.

**Like wise**, **the reference of the commercial antibody for MPO (ab208670, Abcam, UK) have been added to materials and methods section in page 8.**

**Moreover, the immunohistochemistry for MPO was quantitated and added in results section in table 2**

6. The title should be modified, no direct proof that Panax ginseng alleviates TAA-induced liver injury via AGEs modulation is provided. The authors only show a correlation betweed this treatment and a reduction in AGEs levels, as they also find a correlation with increased hepatic antioxidant capacity.

**We appreciate this comment so the title has been modified as suggested by the reviewer to be “****Panax Ginseng Alleviates Thioacetamide-Induced Liver Injury in Ovariectomized Rats: Crosstalk between Inflammation and Oxidative Stress.”**

7. Minor.

“Panax ginseng” should be always written in italics.

**Thank you for this comment.**

**Panax ginseng has been changed into italics allover the manuscript as per the reviewer’s comment.**

**Thank you**
